# Supplementary figures and images for: Genome insights from the identification of a novel Pandoraea sputorum isolate and its characteristics
Source: PLoS One. 2022 Aug 5;17(8):e0272435. doi: 10.1371/journal.pone.0272435 (PMC9355198; doi:10.1371/journal.pone.0272435)

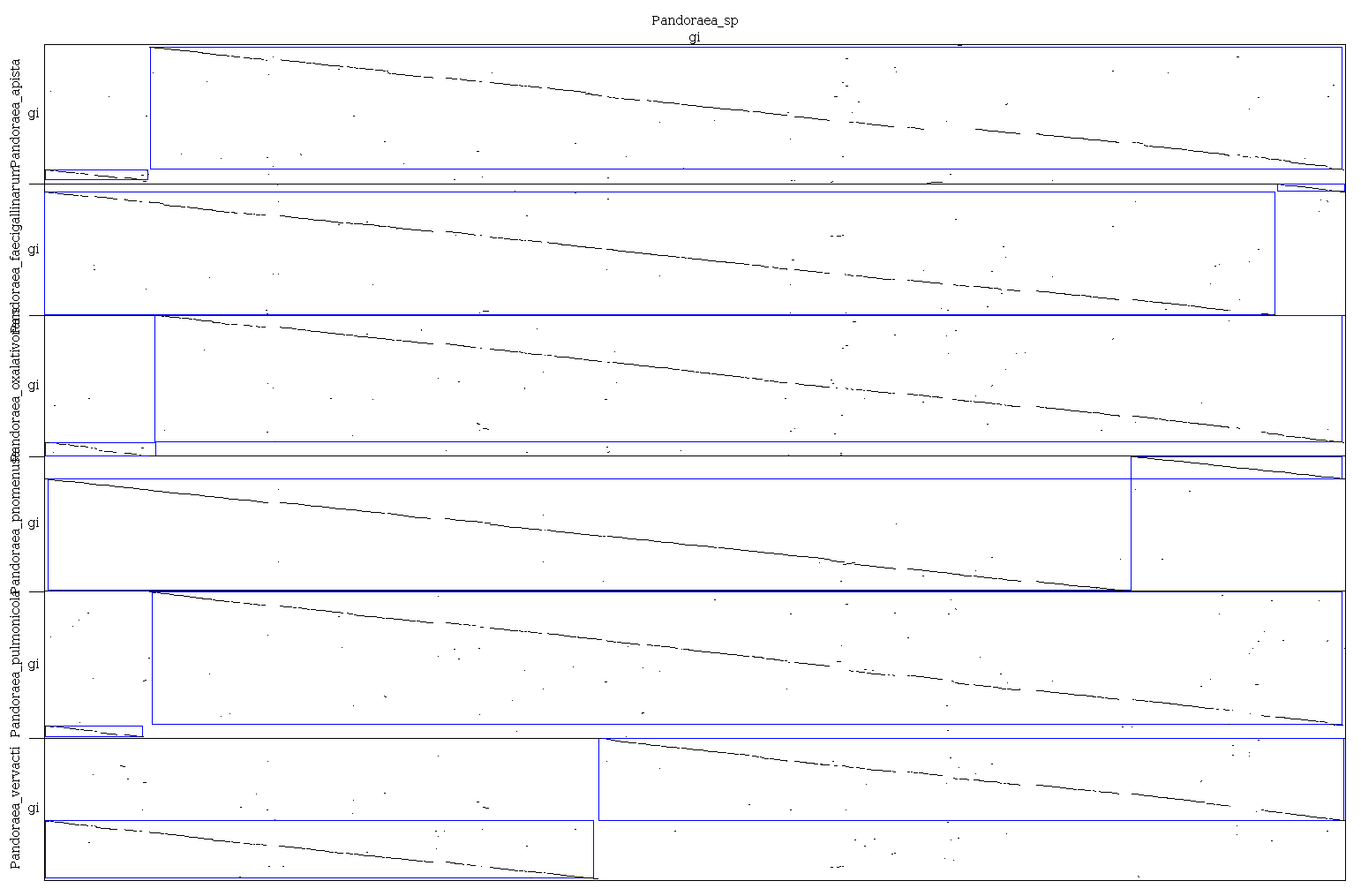


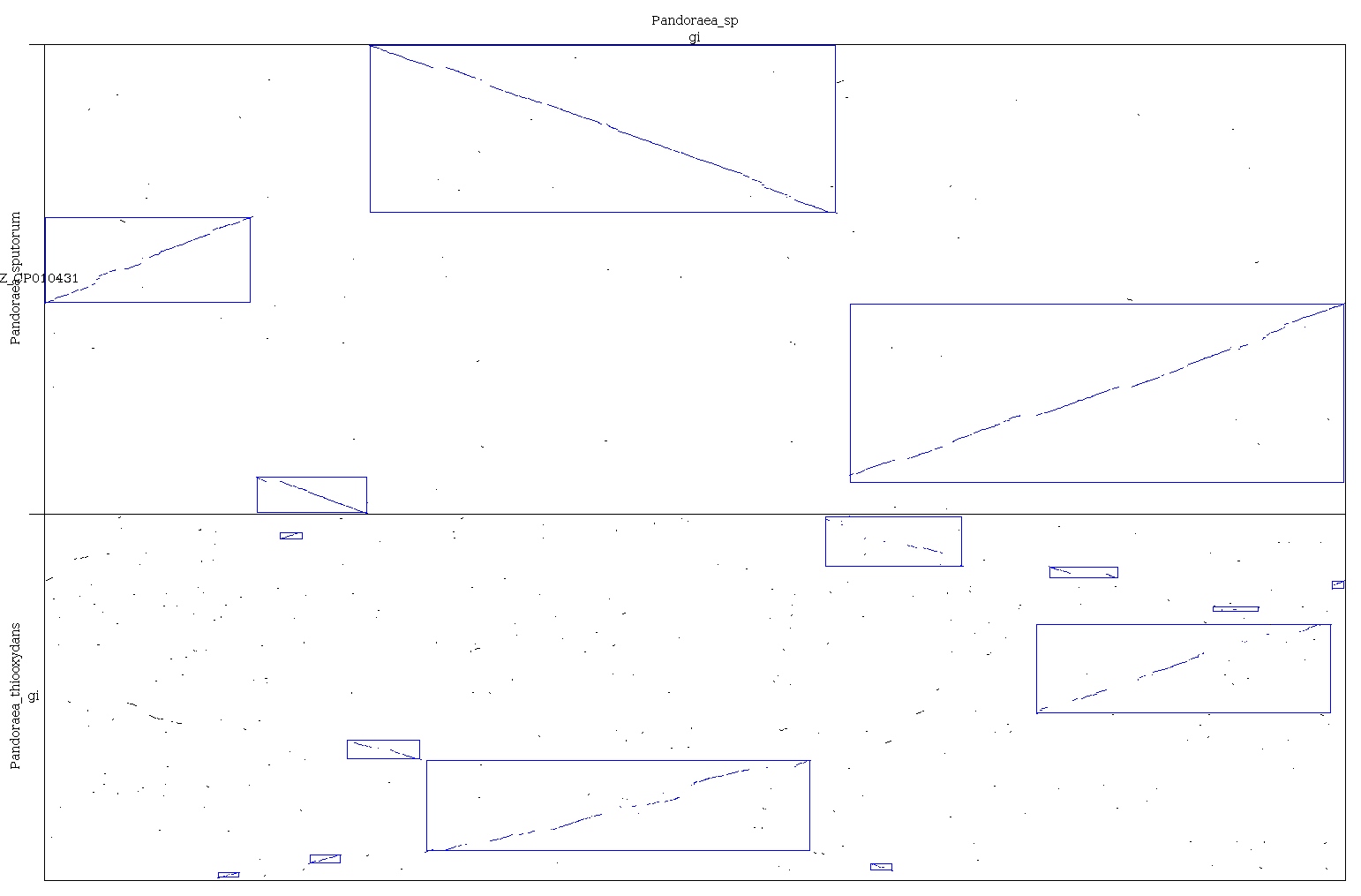


S1 Fig. Diagram of linear genomic organization among *Pandoraea* strains.

Supplement: S1 Fig — (DOC) [file pone.0272435.s001.doc]
