# Supplementary material for: Genome insights from the identification of a novel Pandoraea sputorum isolate and its characteristics
Source: PLoS One. 2022 Aug 5;17(8):e0272435. doi: 10.1371/journal.pone.0272435 (PMC9355198; doi:10.1371/journal.pone.0272435)
Supplement: S2 Fig — Scaffold1, scaffold2, scaffold3, scaffold4, and scaffold5 were the five largest sequences. (DOC) [file pone.0272435.s002.doc]

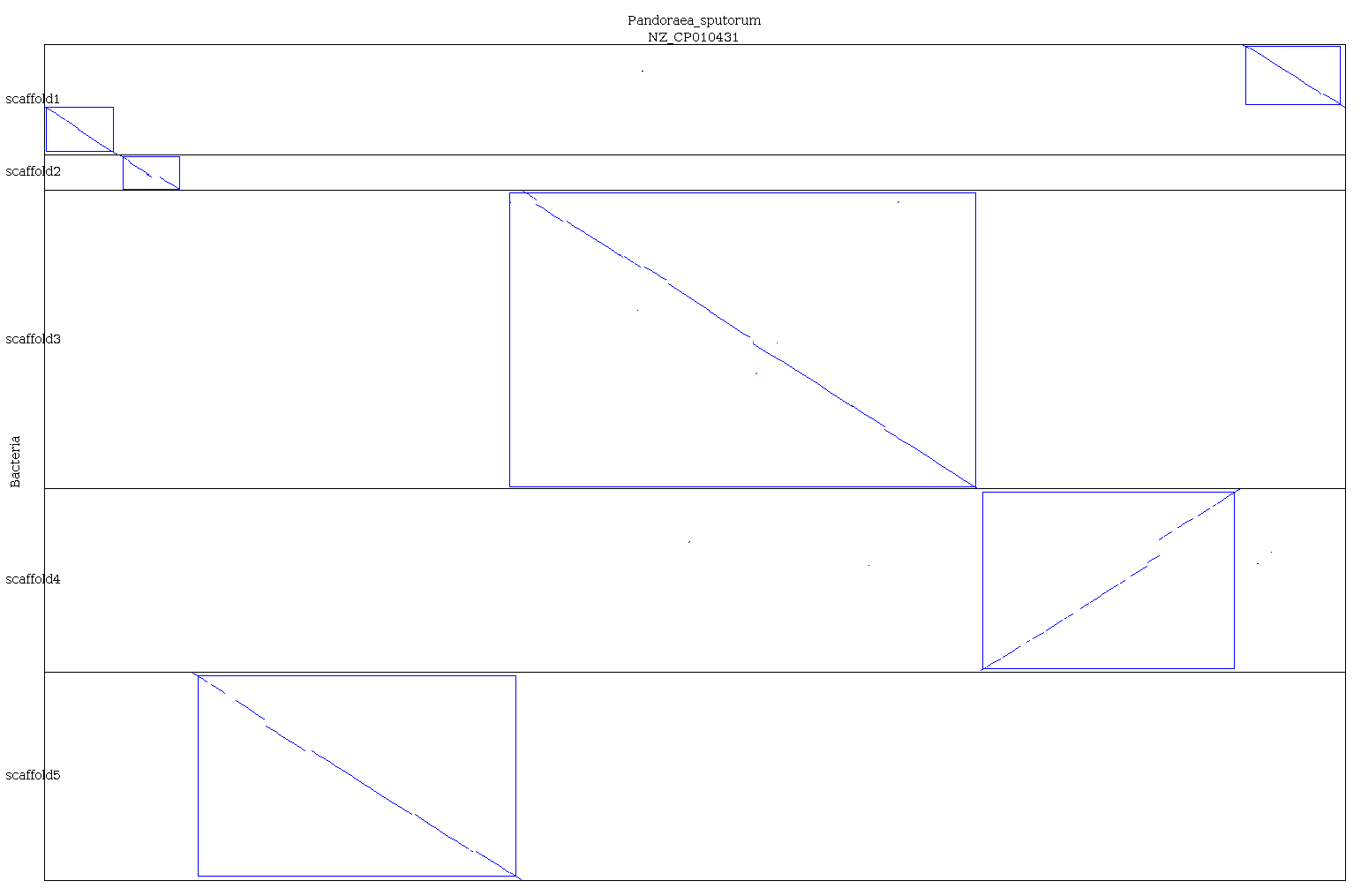


S2 Fig. Diagram of linear genomic organization between *Pandoraea* sp. 892iso and *Pandoraea* *sputorum*. Scaffold1, scaffold2, scaffold3, scaffold4, and scaffold5 were the five largest sequences.
