# Supplementary material for: Genome insights from the identification of a novel Pandoraea sputorum isolate and its characteristics
Source: PLoS One. 2022 Aug 5;17(8):e0272435. doi: 10.1371/journal.pone.0272435 (PMC9355198; doi:10.1371/journal.pone.0272435)
Supplement: Attachment — Submitted filename: renamed_7c11c.docx [file pone.0272435.s004.docx]

**Comment 1.** Please ensure that your manuscript meets PLOS ONE's style requirements, including those for file naming. The PLOS ONE style templates can be found at 
https://journals.plos.org/plosone/s/file?id=wjVg/PLOSOne_formatting_sample_main_body.pdf and https://journals.plos.org/plosone/s/file?id=ba62/PLOSOne_formatting_sample_title_authors_affiliations.pdf

Response:

Thank you for your comments. The format of manuscript has been modified according to the example of PLOS ONE's style.

**Comment 2.** We note that the grant information you provided in the ‘Funding Information’ and ‘Financial Disclosure’ sections do not match. When you resubmit, please ensure that you provide the correct grant numbers for the awards you received for your study in the ‘Funding Information’ section.

Response:

Thank you for your comments. The ‘Financial Disclosure’ has been modified as: Grant funds of No. 2016YFF0203204 and No. 2012BAK11B06 are used in study design, data collection and analysis. The funds of No. 2021HK171 is used for the decision to publish, and preparation of the manuscript.

**Comment 3.** Thank you for stating the following in the Acknowledgments Section of your manuscript:

This work was supported by National Key R&D Programme of China (No. 2016YFF0203204) and National Key Technology Research and Development Programme of China (No. 2012BAK11B06) for G.-M. Z. Scientific research project of General Administration of Customs. P. R. China (2021HK171) for Y. W.

We note that you have provided additional information within the Acknowledgements Section that is not currently declared in your Funding Statement. Please note that funding information should not appear in the Acknowledgments section or other areas of your manuscript. We will only publish funding information present in the Funding Statement section of the online submission form. 
Please remove any funding-related text from the manuscript and let us know how you would like to update your Funding Statement. Currently, your Funding Statement reads as follows:

The funders had no role in study design, data collection and analysis, decision to publish, or preparation of the manuscript.

Please include your amended statements within your cover letter; we will change the online submission form on your behalf.

Response:

Thank you for your comments. The Acknowledgement has been removed in the manuscript.

**Comment 4.** PLOS requires an ORCID iD for the corresponding author in Editorial Manager on papers submitted after December 6th, 2016. Please ensure that you have an ORCID iD and that it is validated in Editorial Manager. To do this, go to ‘Update my Information’ (in the upper left-hand corner of the main menu), and click on the Fetch/Validate link next to the ORCID field. This will take you to the ORCID site and allow you to create a new iD or authenticate a pre-existing iD in Editorial Manager. Please see the following video for instructions on linking an ORCID iD to your Editorial Manager account: https://www.youtube.com/watch?v=_xcclfuvtxQ

Response:

Thank you for your comments. Yes, I have an ORCID iD and use it to log in.

**Comment 5.** Please ensure that you refer to Figure 1 in your text as, if accepted, production will need this reference to link the reader to the figure.

Response:

Thank you for your comments. We have added refer of Figure 1 in the section of Genome assembly, annotation, and validation of protein-coding genes.

**Comment 6.** We note you have included a table to which you do not refer in the text of your manuscript. Please ensure that you refer to Table 1 in your text; if accepted, production will need this refer ence to link the reader to the Table.

Response:

Thank you for your comments. We have added refer of Table 1 in the section of Genome assembly, annotation, and validation of protein-coding genes.

**Comment 7.**  Thank you for submitting the above manuscript to PLOS ONE. During our internal evaluation of the manuscript, we found significant text overlap between your submission and the following previously published works, some of which you are an author.

- https://journals.asm.org/doi/10.1128/AAC.01112-15
- https://www.cell.com/heliyon/fulltext/S2405-8440(21)00422-9?_returnURL=https%3A%2F%2Flinkinghub.elsevier.com%2Fretrieve%2Fpii%2FS2405844021004229%3Fshowall%3Dtrue
- https://pubmed.ncbi.nlm.nih.gov/15571809/

We would like to make you aware that copying extracts from previous publications, especially outside the methods section, word-for-word is unacceptable. In addition, the reproduction of text from published reports has implications for the copyright that may apply to the publications.

Please revise the manuscript to rephrase the duplicated text, cite your sources, and provide details as to how the current manuscript advances on previous work. Please note that further consideration is dependent on the submission of a manuscript that addresses these concerns about the overlap in text with published work.

Response:

Thank you for your comments.

We have revised the section of Intrinsic Carbapenem-Hydrolyzing Oxacillinases, which has overlap with the publication https://journals.asm.org/doi/10.1128/AAC.01112-15, and the section of Comparative genomic analysis, which has overlap with the publication of https://pubmed.ncbi.nlm.nih.gov/15571809/. The second publication mentioned above is our own work. Some modifications were made in the writing

Reviewer #1: 
Major comments:

**Comment 1.** While the authors have been quite systematic in their comparison of strain *Pandoraea* sp. 892iso with other species at the genomic level, they have not included a large number of species which have been added to this genus in the last few years.

Response:

Thank you for your comments. We have scanned the genome information of the genus of *Pandoraea*, especial of the genomic data published in recent years, including *P. commovens, P. anapnoica, P. aquatica, P. captiosa, P. anhela, P. capi, P. morbifera, P. horticolens, P. communis, P. fibrosis, P. bronchicola, P. cepalis, Pandoraea* sp. XY-2, *P. soli, P. terrigena, P. eparura, P. nosoerga, P. iniqua, P norimbergensis* and *P. terrae*. The corresponding text and figures have all been revised after the genome data added.

**Comment 2.** In addition, while the analysis is thorough, a considerable portion of their manuscript focuses on the description of where this strain sits in the taxonomy of this genus and this seems to be out of proportion with respect to the final conclusion i.e. that this strain belongs to *P. sputorum*. It would be better if the authors focused less on the where their strain fits in this genus and more on the interesting aspects concerning their quorum sensing and oxacillinase gene comparisons and their MLSA typing approach.

Response:

Thank you for your comments. We have updated more *Pandoraea* species data to be included in the relevant analysis, such as, quorum sensing, oxacillinase gene, *rpoB* gene and MLSA.

Minor comments:
**Comment 3.** Please avoid using the word “strain”, unless required, or else preface it with “type” or “reference” where appropriate (for example in the legend for figure 3).

Response:

Thank you for your comments. We replaced the strains in several places with type.

**Comment 4.** Title: This should be more informative.

Response:

Thank you for your comments. The title has been modified as Genome insights from the identification of a novel *Pandoraea sputorum* isolate and its characteristics

**Comment 5.** Abstract:
Line 13-would be useful to explain what *Phytophthora rubi* is.

Line 15: Would be useful to clarify which *Pandoraea* species you are referring to.

Response:

Thank you for your comments. This sentence has been modified as: In this study, we sequenced a bacteria isolate *Pandoraea* sp. 892iso isolated from a *Phytophthora rubi* strain which is an important plant pathogenic oomycete, identified through genome and combined the data with existing genomic data from other 28 the genus of *Pandoraea* species.

**Comment 6.** Line 21: Please make it clear which Oomycetes strain you are referring to.

Response:

Thank you for your comments. This sentence has been modified as: It is speculated that *Pandoraea* sp. 892iso is the endosymbiont of the Oomycetes strain of *Phytophthora rubi*.

**Comment 7.**

Introduction:
Line 27: Use “genus” rather than “species”

Response:

Thank you for your comments. It has been modified.

**Comment 8.** Line 30: Please re-structure this sentence as it is unclear.

Response:

Thank you for your comments. This sentence has been modified as: Some species in this genus were once identified closest to *Burkholderia cepacia* complex (Bcc), *Ralstonia pickettii*, or *Ralstonia paucula* based on phenotype.

**Comment 9.** Line 33: Many additional species have been added to this genus in the last few years. Please see Peeters et al., (2019) Comparative Genomics of Pandoraea, a Genus Enriched in Xenobiotic Biodegradation and Metabolism.Front. Microbiol. 10:2556 and the following for additional species: Anandham et al., 2010; Sahin et al., 2011; Jeong et al., 2016). Please update your manuscript with this information.

Response:

Thank you for your comments. The data in these references has been added in the analysis of all parts, and the references all been added.

**Comment 10.** Line 40: “Further”
Line 67: “were identified through whole genome sequencing”
Lines 68 and 75: Please use “isolate” rather than strain
Response:

Thank you for your comments. All these Spelling mistakes have been corrected.

Materials and Methods:
**Comment 11.** Line 90: Do you mean “calculations” rather than “calculate”?

Response:

Yes, it has been corrected.

**Comment 12.** Line 95 and 100: please clarify what you mean by “11 species”.

Response:

It means all the well genome data of the genus of *Pandoraea.*

**Comment 13.**

Results: Line 114: Which Pandoraea sputorum isolate are you referring to? If it is Pandoraea sp. 892iso It may be better to use the name of the strain at this stage as the evidence for the WGS-based identification has not yet been presented.

Response:

This sentence has been modified as: The genome of the *Pandoraea* sp. 892iso isolate was assembled from sequencing data generated by HiSeq 2000 by SOAPDenovo2 assembler.

**Comment 14.** Line 134: It would be helpful if the authors included some details about the types of single copy genes chosen for their generating their phylogenetic tree, with the genes listed in a table, or in supplementary information.

Response:

Thank you for your comments. This list of single copy genes has been added as supplementary Table 1.

**Comment 15.** Lines 183-188: The decision to choose these particular house-keeping genes should be explained.

Response:

Thank you for your comments. These house-keeping genes used for MLSA analysis were from the reference 40 and 42.

**Comment 16.**Line 207: A much shorter title to this section should be chosen.

Response:

Thank you for your comments. This title has been modified as: Some special genes among *Pandoraea* sp.

**Comment 17.** Figures and Tables:
Figure 2: The legend for this figure needs further information about the methods used to generate this data.

Response:

Thank you for your comments. It has been added.

**Comment 18.** Table 1: Please clarify the accession numbers and details for the P. pulmonicola, P. sputorum genomes used, and also for the other species for which this information had not been provided.

Response:

Thank you for your comments. The accession numbers of all strains in Table 2 have been added.

**Comment 19.** Table 2: This table needs some description in the legend, and further details in the main text to explain what it depicts.

Response:

Thank you for your comments. The information of Table 2 has been added.
