# Supplementary material for: Genome insights from the identification of a novel Pandoraea sputorum isolate and its characteristics
Source: PLoS One. 2022 Aug 5;17(8):e0272435. doi: 10.1371/journal.pone.0272435 (PMC9355198; doi:10.1371/journal.pone.0272435)
Supplement: Attachment — Submitted filename: 4th-Response to editor.doc [file pone.0272435.s006.doc]

**Comment 1.** Figures 4 and 5 need to be improved so they can be published as supplementary figures. The manuscript has to be revised to be adapted and the figures numbered according.

Response:

Thank you for your comments.

Figures 4 and 5 have been removed as supplementary figures, the number of figures in the manuscript have also been adapted.
